# MRI radiomics combined with machine learning for diagnosing mild cognitive impairment: a focus on the cerebellar gray and white matter

**DOI:** 10.3389/fnagi.2024.1460293

**Published:** 2024-10-04

**Authors:** Andong Lin, Yini Chen, Yi Chen, Zhinan Ye, Weili Luo, Ying Chen, Yaping Zhang, Wenjie Wang

**Affiliations:** ^1^Department of Neurology, Municipal Hospital Affiliated to Taizhou University, Taizhou, China; ^2^Department of Radiology, The First Affiliated Hospital of Dalian Medical University, Dalian, Liaoning Province, China; ^3^Department of Pharmacy, Taizhou Central Hospital (Taizhou University Hospital), Taizhou, China; ^4^Department of Neurology, Taizhou Central Hospital (Taizhou University Hospital), Taizhou, China

**Keywords:** mild cognitive impairment, machine learning, radiomics, cerebellum, gray matter, white matter

## Abstract

**Objective:**

Mild Cognitive Impairment (MCI) is a recognized precursor to Alzheimer’s Disease (AD), presenting a significant risk of progression. Early detection and intervention in MCI can potentially slow disease advancement, offering substantial clinical benefits. This study employed radiomics and machine learning methodologies to distinguish between MCI and Normal Cognition (NC) groups.

**Methods:**

The study included 172 MCI patients and 183 healthy controls from the Alzheimer’s Disease Neuroimaging Initiative (ADNI) database, all of whom had 3D-T1 weighted MRI structural images. The cerebellar gray and white matter were segmented automatically using volBrain software, and radiomic features were extracted and screened through Pyradiomics. The screened features were then input into various machine learning models, including Random Forest (RF), Logistic Regression (LR), eXtreme Gradient Boosting (XGBoost), Support Vector Machines (SVM), K Nearest Neighbors (KNN), Extra Trees, Light Gradient Boosting Machine (LightGBM), and Multilayer Perceptron (MLP). Each model was optimized for penalty parameters through 5-fold cross-validation to construct radiomic models. The DeLong test was used to evaluate the performance of different models.

**Results:**

The LightGBM model, which utilizes a combination of cerebellar gray and white matter features (comprising eight gray matter and eight white matter features), emerges as the most effective model for radiomics feature analysis. The model demonstrates an Area Under the Curve (AUC) of 0.863 for the training set and 0.776 for the test set.

**Conclusion:**

Radiomic features based on the cerebellar gray and white matter, combined with machine learning, can objectively diagnose MCI, which provides significant clinical value for assisted diagnosis.

## Introduction

Alzheimer’s disease (AD) is a chronic neurodegenerative disorder and the most prevalent form of dementia, characterized by progressive memory impairment accompanied by at least one of the following symptoms: aphasia, apraxia, agnosia, or executive dysfunction (Soria Lopez et al., 2019). The onset and progression of AD are continuous processes, with Mild Cognitive Impairment (MCI) considered a pre-clinical stage of AD (Anderson, 2019; Jongsiriyanyong and Limpawattana, 2018), posing a high risk of transition to AD (Jacobs et al., 2018). Therefore, early diagnosis and timely treatment of MCI can delay disease progression, offering significant clinical value in improving prognosis (Lim, 2016; Suh et al., 2020).

Traditionally, the cerebellum has been viewed as a crucial structure for orchestrating motor functions, with little to no association with AD onset or progression (Grodd et al., 2005; Hoenig et al., 2018). However, emerging studies suggest that beyond its role in fine motor control, the cerebellum may also contribute to cognitive processes and emotional expression (Schmahmann, 2010; Tedesco et al., 2011; Li et al., 2023). Concurrently, recent histopathological investigations have revealed neurodegenerative and neuropathological alterations in the cerebellum of AD patients. These changes encompass amyloid plaque accumulation, notable reductions in Purkinje cell density, and atrophy affecting both the molecular and granular cell layers. Furthermore, individuals with cerebellar damage often present with a range of cognitive impairments, collectively termed Cerebellar Cognitive Affective Syndrome (CCAS). This syndrome encompasses executive dysfunction, visual–spatial deficits, language processing challenges, and emotional regulation disturbances, reinforcing the proposition that the cerebellum is implicated in cognitive function modulation (Muenchhoff et al., 2014; Yang et al., 2020).

fMRI is a non-invasive imaging technique that measures blood oxygen level-dependent signals during brain activity, namely changes in cerebral blood flow. This helps reveal the connections and activity patterns between different regions of the brain, which is crucial for studying the pathophysiological mechanisms of AD and MCI. Research has shown that compared to healthy elderly controls, increased functional magnetic resonance imaging activity in the hippocampus and/or entorhinal cortex at baseline in MCI patients may indicate a higher likelihood of cognitive decline (Dickerson et al., 2004). Studies by Fanyu Tang and others have demonstrated through fMRI methods that cortical-cerebellar functional connectivity (FC) is significantly impaired and differently distributed in MCI and AD patients (Tang et al., 2021). However, fMRI also has drawbacks such as long scanning times, poor reproducibility, and numerous artifacts. In contrast, structural Magnetic Resonance Imaging (sMRI) has excellent reproducibility, which benefits long-term follow-up studies and monitoring disease progression or treatment effects. Hippocampal or medial temporal lobe atrophy measured on sMRI has been incorporated into the National Institute on Aging and the Alzheimer’s Association (NIA-AA) recommendations for the diagnosis of MCI caused by Alzheimer’s disease as a marker of neuronal damage (Albert et al., 2011).

Early identification of MCI is beneficial for timely intervention and treatment. Although clinical assessment can diagnose MCI, it may not be sensitive to early subtle changes. There exists a pressing need for objective and highly sensitive diagnostic methods in clinical settings. Radiomics is the process of extracting numerous imaging features that delineate disease characteristics from medical images through high-throughput analysis (Lambin et al., 2017). The integration of radiomics with machine learning represents a significant advancement in the field of medical image analysis. This combination provides powerful tools and methods for improving the speed and precision of diagnoses, developing personalized treatment plans, and conducting in-depth research on complex diseases (Sharma et al., 2023; Yang et al., 2023).

Presently, the primary focus of radiomics and machine learning in AD is the hippocampus (Zhang et al., 2011; Luk et al., 2018), which has demonstrated commendable accuracy rates. Nonetheless, research concerning the cerebellum remains comparatively limited. Although the technical advantages of using cerebellar characteristics to assist in the diagnosis of MCI are not yet clear, we want to explore the potential abnormalities in patients with MCI from this new perspective of the cerebellum. Therefore, in this study, we extracted radiomic features based on cerebellar gray and white matter, and subsequently established a model to facilitate the rapid differentiation between MCI and HC. In this study, we presume that the radiomic features derived from both the cerebellar gray and white matter possess consistent diagnostic efficacy for MCI. By utilizing model visualization techniques, we aim to further evaluate and compare their respective importance.

## Materials and methods

### Subjects

All sample data for this study were obtained from the Alzheimer’s Disease Neuroimaging Initiative (ADNI; http://adni.loni.usc.edu/) database. Established in 2003, ADNI primarily aims to monitor the progression of MCI and AD by incorporating various neuropsychological assessments, neuroimaging techniques, and other biomarkers. We chose the data from ADNI 1, setting the filtering criteria to 3D T1WI image data obtained from baseline period scans of MCI and CN. This study encompassed a total of 355 instances of 3D-T1 weighted MR structural images, which included 172 MCI patients and 183 healthy individuals. All participants from baseline period were subjected to stringent exclusion criteria. The statistical information related to this sample data was presented in Table 1. As per the ADNI protocol, the diagnostic criteria for MCI should include: (a) a subjective report of memory concerns, objective memory loss, absence of dementia, and preserved daily activities; (b) a Mini-Mental State Examination (MMSE) score of ≥24. Further details regarding the diagnostic criteria can be found on the ADNI website.

**Table 1:** Demographic data of the MCI and HC groups.

	HC (*n* = 183)	MCI (*n* = 172)	Statistic	*p*
Age/year	76.00 (73.00, 79.00)	76.00 (71.00, 81.00)	*Z* = −0.17	0.862
Sex, *n* (%)			χ^2^ = 14.44	<0.001
Male	88 (48.09)	117 (68.02)		
Female	95 (51.91)	55 (31.98)		

HC, Healthy controls; MCI, Mild cognitive impairment; *Z*, Mann–Whitney test; χ^2^, Chi-square test.

### Image preprocessing

For all participants, image acquisition was performed using the 3D-T1-MPRAGE or equivalent protocols with slimly different resolutions was used. The ADNI website provides all detailed imaging parameters. For scanner 1 (Siemens Medical Solutions, 3.0T), scanning parameters are as follows: repetition time (TR) = 2300.0 ms, echo time (TE) = 3.0 ms, matrix = 240 × 256 × 176. For scanner 2 (General Electric Healthcare, 3.0 T), scanning parameters are: TR = 7.7–7.0 ms, TE = 3.1–2.8 ms, matrix = 256 × 256 × 196. For scanner 3 (Philips Medical Systems, 3.0 T), Mr. imaging data were acquired with the following parameters: TR = 6.8 ms, TE = 3.1 ms, matrix = 256 × 256 × 170. The slice thickness for the three different scanners was either 1.0 or 1.2 mm, with a slice gap of 0 mm.

### Image preprocessing and radiomics feature extraction

Before extracting MRI radiomics features, all data undergo preprocessing, adhering to the Image Biomarker Standardization Initiative (IBSI) (Zwanenburg et al., 2020). The specific steps of image preprocessing are as follows:

1. Format conversion: Use dcm2niix from the MRIcron^1^ software to convert MRI images from dicom format to Neuroimaging Informatics Technology Initiative (NIFTI, nii) format for subsequent processing.
2. Segmentation: Automated segmentation of the cerebellum is performed using volBrain,^2^ a powerful high-precision automated brain segmentation tool, which can extract white matter and gray matter of the cerebellum. The exclusive CERES is an automatic segmentation and extraction of white matter and gray matter in the cerebellum from MRI.
3. Image normalization: Prior to feature extraction, it is crucial to resample the MRI images using Python in order to standardize voxel sizes to 1 × 1 × 1 mm^3^. Subsequently, grayscale values should be discretized with a bin width of 25.

After image preprocessing is completed, use Pyradiomics in Python^3^ to extract features from all white and gray matter. This study extracts 833 radiomics features from each white and gray matter, including 14 shape features, 18 first-order features, 22 Gray-Level Co-occurrence Matrix (GLCM) features, 14 Gray-level dependence matrix (GLDM) features, 16 Gray level size zone matrix (GLSZM) features, 16 Gray level run-length matrix (GLRLM) features, five Neighboring gray tone difference matrix (NGTDM) features, and 728 wavelet features. Details are provided in Supplementary Table S1.

### Radiomics feature data preprocessing

Before conducting radiomics feature selection, it is necessary to preprocess the data. Due to the different orders of magnitude of the data in radiomics features, this study adopts the *Z*-score standardization method to transform the original image pixel values through the formula “*Z* = [*x* − mean (*x*)]/std. (*x*),” that is, calculating the mean and standard deviation of each radiomics feature in the training set to obtain the *Z*-score value.

### Radiomics feature selection

To obtain relatively stable radiomic features, we randomly selected 20 samples for two rounds of segmentation and retained the features with an Intra-class Correlation Coefficient (ICC) greater than 0.8 for further calculations. The remaining features are used for further filtering and modeling. Firstly, the radiomic features are subjected to Mann–Whitney U test for statistical verification and feature selection, retaining only those radiomic features with a *p* value <0.05. If the correlation coefficient between two features exceeded 0.90, the feature exhibiting a higher Pearson correlation with other features was eliminated. To maximally preserve the ability to characterize features, we adopt a greedy recursive deletion strategy for feature selection, which involves deleting the feature with the highest redundancy in the current set each time (the advantage of this method is that it efficiently pinpoints the most informative features by sequentially discarding the least significant ones, resulting in more streamlined models with potentially enhanced performance). Subsequently, the Least Absolute Shrinkage and Selection Operator (LASSO) is used to construct features. Based on the tuning weight “*λ*,” LASSO shrinks all regression coefficients to zero, precisely setting the coefficients of many irrelevant features to zero. To find the optimal “λ” value, this study employed 5-fold cross-validation and the one standard error rule, ultimately selecting the “λ” value that produced the smallest cross-validation error. Features with non-zero coefficients are retained for regression model fitting and combined into radiomic signatures. Radiomic scores are calculated for each patient.

### Model establishment and verification

**Figure 1:**
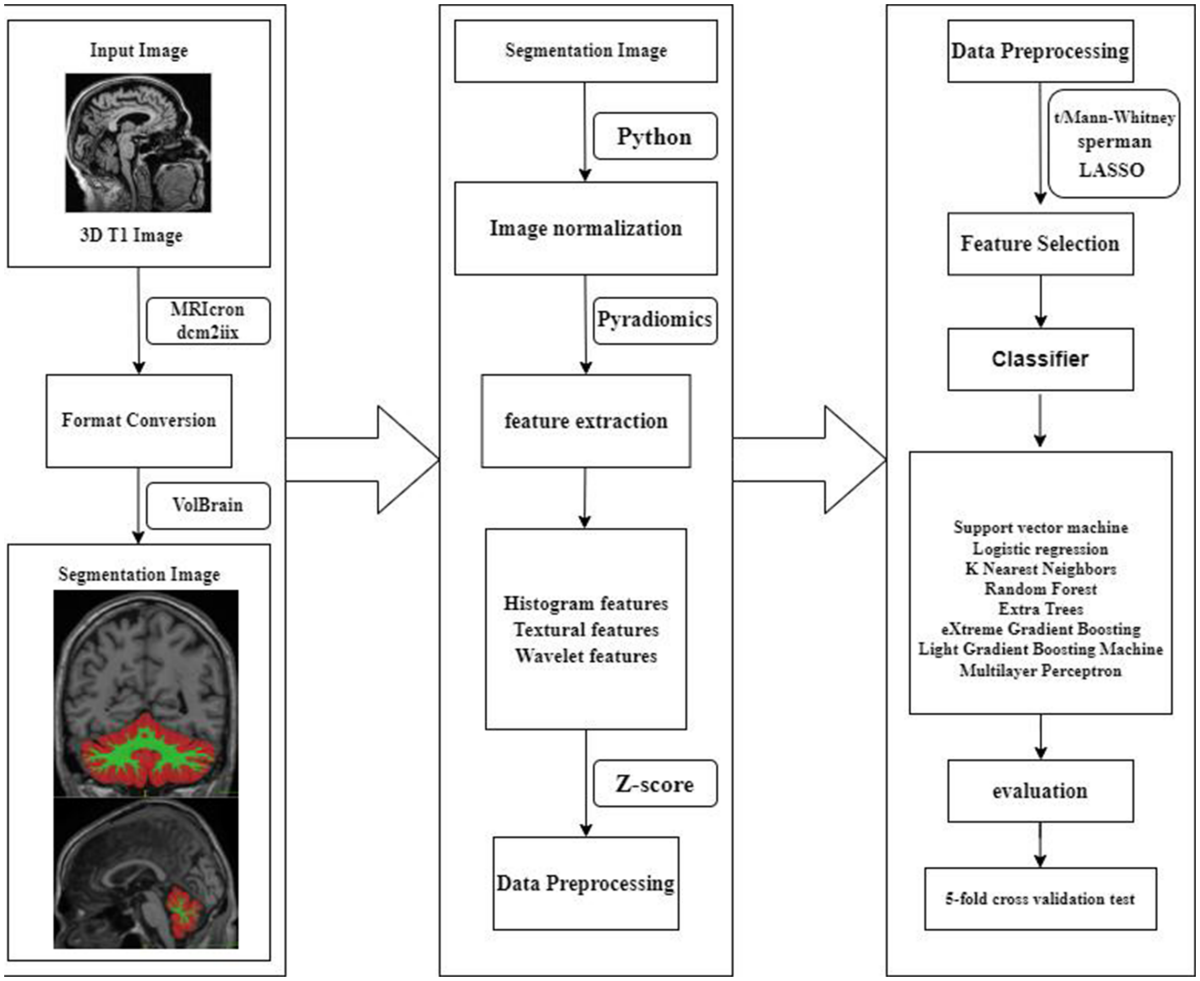
A schematic diagram for the whole radiomics and machine learning pipeline.

Before establishing the model, all patients were randomly divided into a training group and a test group at an 8:2 ratio. We input the final features into machine learning models such as Random Forest, Logistic Regression (LR), eXtreme Gradient Boosting (XGBoost), Support Vector Machines (SVM), K Nearest Neighbors (KNN), Extra Trees, Light Gradient Boosting Machine (LightGBM), and Multilayer Perceptron (MLP). Each model underwent 5-fold cross-validation for penalty parameter optimization to construct radiomics models. The flowchart of this study was shown in Figure 1.

### Statistical analysis

The model’s predictive performance is assessed using the receiver operating characteristic curve (ROC), which involves calculating the AUC and its 95% confidence interval (CI), as well as accuracy, sensitivity, and specificity. All statistical analyses are performed using Python software (version 3.9.7, https://www.python.org/), with “Scikit-Learn” for machine learning, and “matplotlib” and “sklearn” for plotting correlation heatmaps, ROC curves, and decision curves.

## Results

### The baseline characteristics of the patients

Table 2 provided a summary of the baseline clinical characteristics for 355 patients, divided into a training set (*N* = 284) and a test set (*N* = 71). The combined training and test sets include 138 cases (48.6%) and 34 cases (45.9%) of patients with mild cognitive impairment, respectively. A significant difference in gender distribution is observed between the normal control and mild cognitive impairment groups within the training set (*p* < 0.001).

**Table 2:** Demographic data of the test and training groups.

	Training set (*n* = 284)			Test set (*n* = 71)		
	HC (*n* = 146)	MCI (*n* = 138)	*p* value	HC (*n* = 37)	MCI (*n* = 34)	*p* value
Age (years)	76.00 (73.00, 80.00)	76.00 (72.00, 81.75)	0.860	75.73 ± 4.54	75.47 ± 6.15	0.842
Gender, *n* (%)			<0.001			0.718
Male	70 (47.95)	99 (71.74)		18 (48.65)	18 (52.94)	
Female	76 (52.05)	39 (28.26)		19 (51.35)	16 (47.06)	
MMSE	29.00 (29.00, 30.00)	27.00 (26.00, 28.00)	<0.001	29.30 ± 0.81	26.94 ± 1.89	<0.001

### Model establishment

Feature selection is scheduled after the completion of feature preprocessing. For detailed information on the results of feature preprocessing, please refer to the Supplementary material. A portion of the samples underwent ICC tests through two repetitive segmentations, ultimately leaving 1643 radiomics features, including 210 original features and 1433 wavelet features. The proportion of various radiomics features was shown in Supplementary Figure S1. Features with statistically significant differences between groups were retained through hypothesis testing, leaving a total of 352 radiomics features, among which there are 46 original features and 306 wavelet features. Through Spearman screening (with a threshold set at 0.9), 142 radiomics features were obtained, including 18 original features and 124 wavelet features.

**Figure 2:**
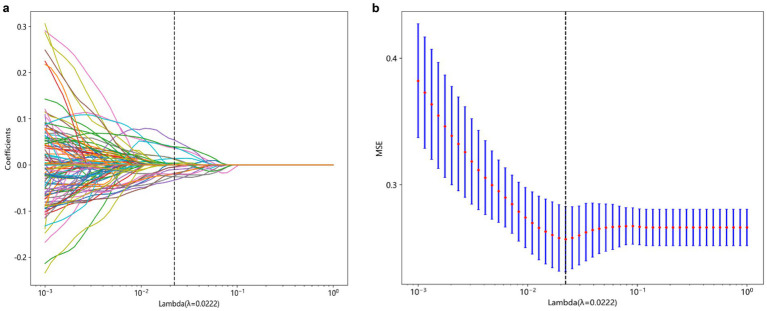
Process of feature selection. The parameters are screened based on lambdamin, that is, when the mean value of the error is the minimum **(A)**. Radiomics features were selected by the LASSO logistic regression model **(B)**. LASSO, Least absolute shrinkage and selection operator.

**Figure 3:**
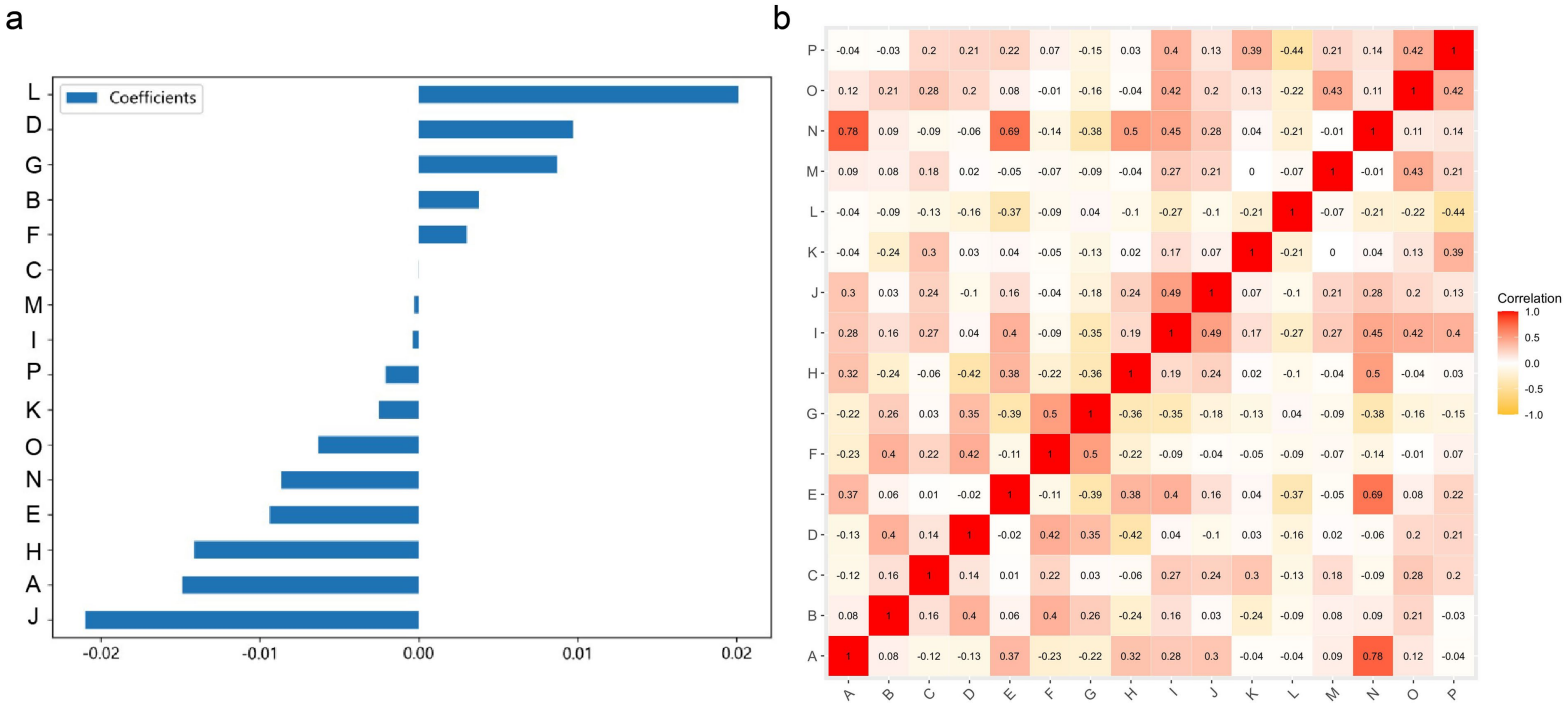
A graph of the relationships between 16 radiomic features used for modeling. **(A)** The weight distribution of radiomic features. **(B)** A heatmap of the correlations between radiomic features.

This study utilized the LASSO to further select features (Figure 2A). Features with non-zero coefficients were retained for regression model fitting (Figure 2B), and Radiomics scores (Rad_score) were calculated for each patient (in Supplementary material). The serial numbers corresponding to the 16 radiomics features included in the modeling were shown in Table 3, their weight distribution was shown in Figure 3A, and the correlation heatmap was shown in Figure 3B.

**Table 3:** Sixteen imaging genomics characteristics included in the modeling.

Index	feature
A	GM_wavelet_HLH_glrlm_RunVariance
B	WM_wavelet_LLL_firstorder_10Percentile
C	WM_original_glszm_GrayLevelNonUniformity
D	WM_wavelet_HLH_glszm_SmallAreaHighGrayLevelEmphasis
E	GM_original_glrlm_RunEntropy
F	WM_wavelet_HLL_firstorder_Maximum
G	WM_wavelet_HLL_firstorder_Skewness
H	GM_wavelet_LHH_glrlm_RunEntropy
I	GM_wavelet_LHL_glrlm_RunLengthNonUniformity
J	WM_original_shape_LeastAxisLength
K	WM_wavelet_LLL_ngtdm_Contrast
L	GM_wavelet_LLL_glcm_ClusterShade
M	GM_wavelet_HHH_glszm_SizeZoneNonUniformity
N	GM_wavelet_HLH_glrlm_RunEntropy
O	GM_wavelet_LHH_glszm_SizeZoneNonUniformity
P	WM_wavelet_LLH_firstorder_RootMeanSquared

### Machine learning algorithm

**Figure 4:**
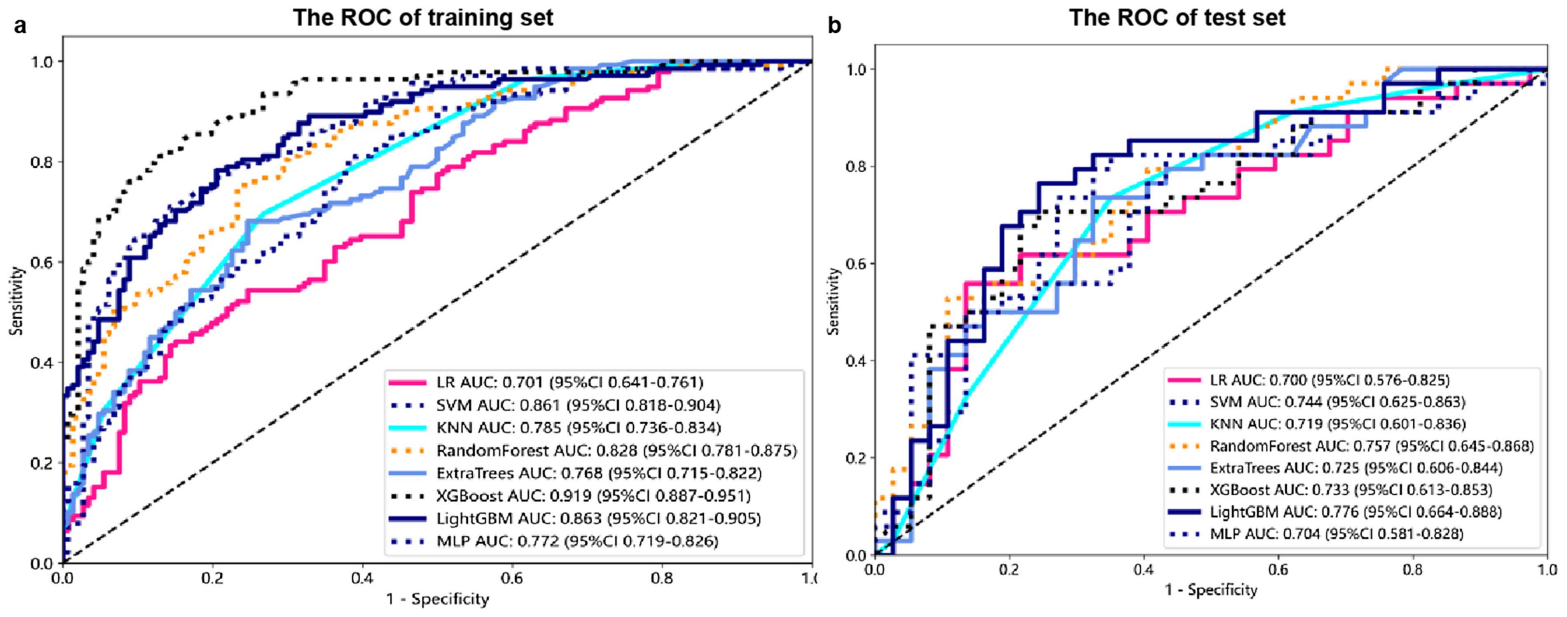
Receiver Operating Characteristic (ROC) curves for each model on the training **(A)** and test sets **(B)**.

**Figure 5:**
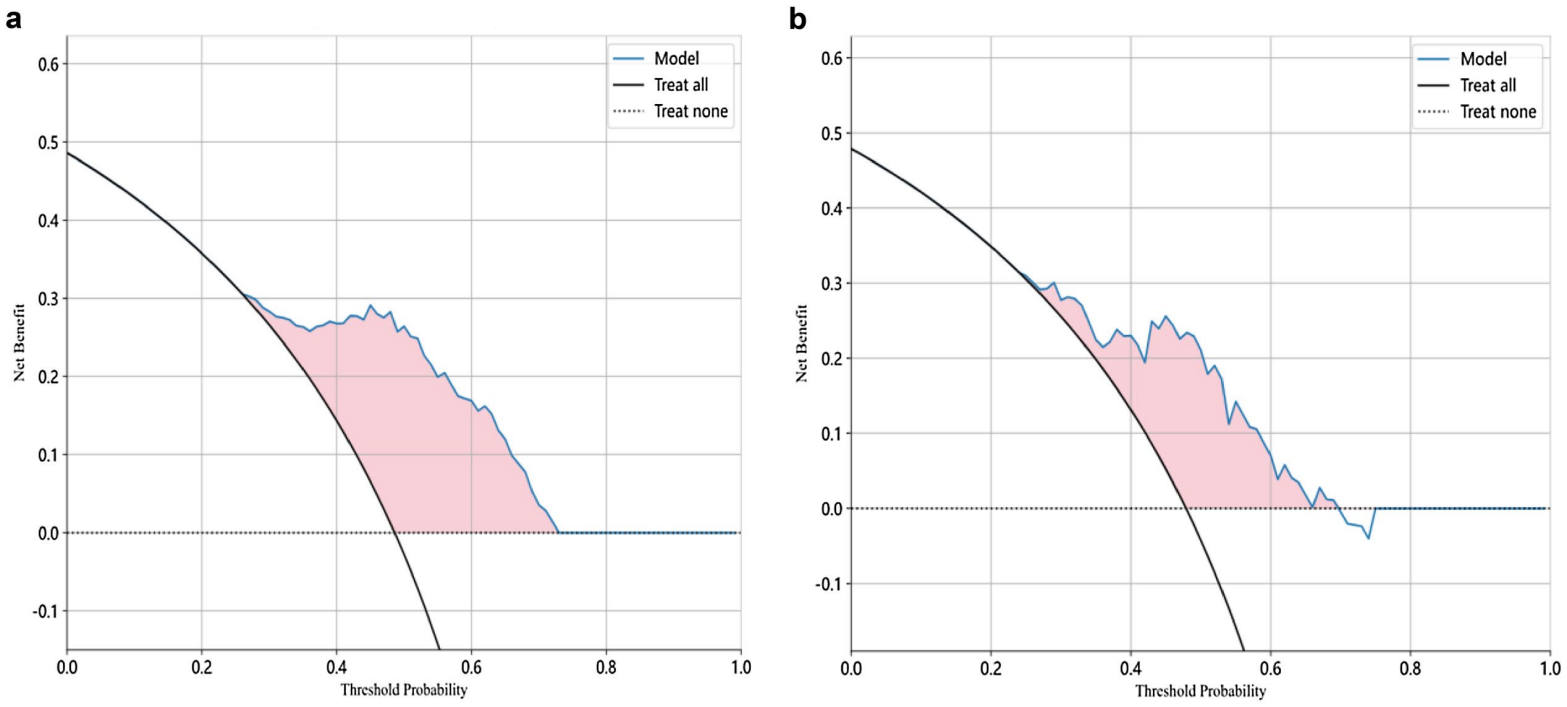
Decision Curve Analysis (DCA) curve of the LightGBM model for both training and test sets.

**Figure 6:**
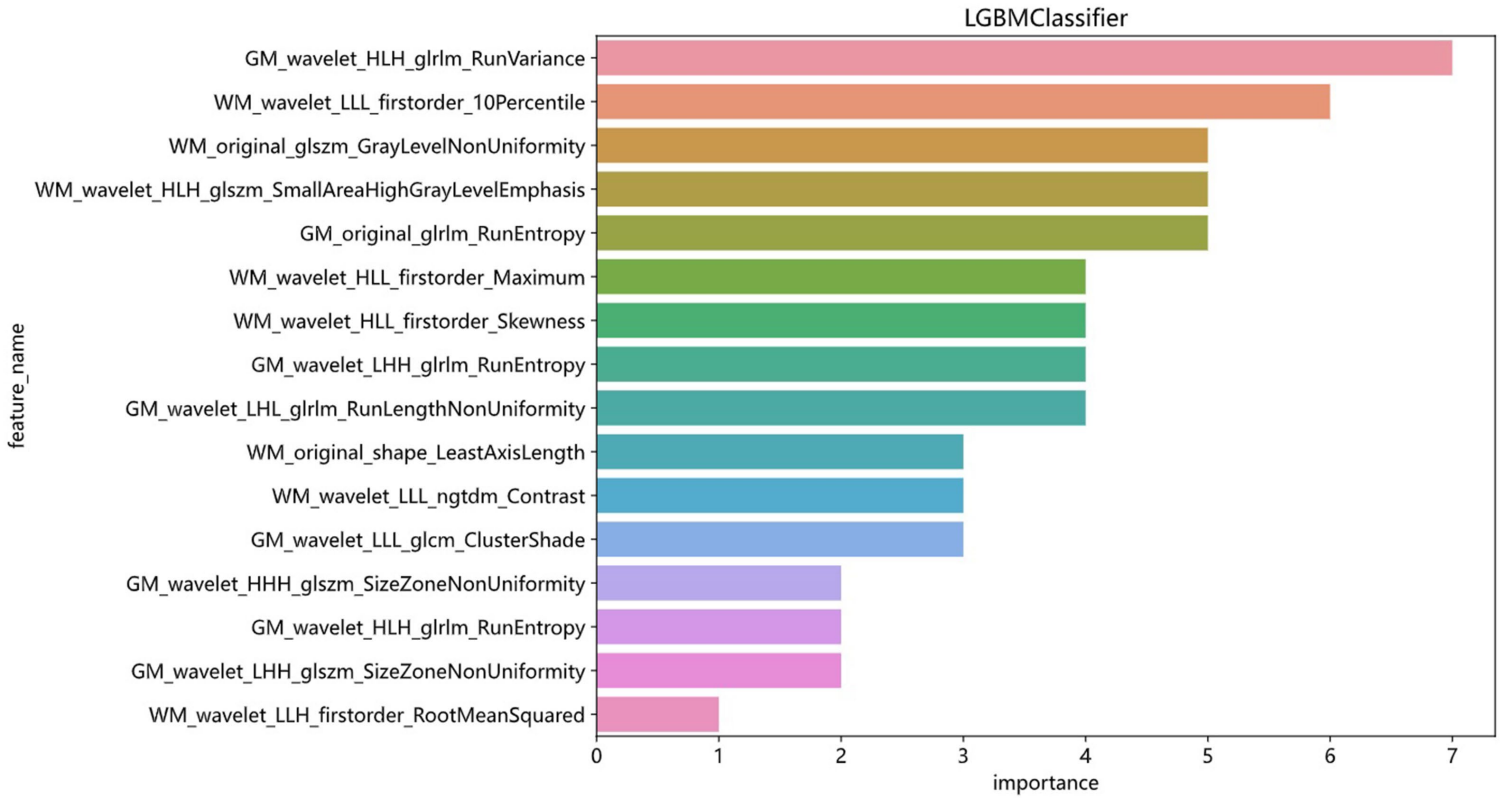
Ranking of the importance of 16 radiomics features in the LightGBM model.

The modeling process incorporated various machine learning algorithms, including Random Forest, Logistic Regression, eXtreme Gradient Boosting (XGBoost), Support Vector Machines (SVM), K-Nearest Neighbors (KNN), Extra Trees: Extremely Randomized Trees, Light Gradient Boosting Machine (LightGBM), and Multilayer Perceptron (MLP). These models were fine-tuned to attain optimal solutions. Table 4 delineated the performance metrics of each algorithm on the training and test sets. Figure 4 depicted the ROC curves for each model on both sets. After comparing multiple indicators in the table, the considered to be the most effective. The DCA curves of the model on the training set and test set are shown in Figure 5. The importance ranking of the 16 radiomic features in the LightGBM model was displayed in Figure 6. Notably, among these 16 features in the LightGBM prediction model, eight pertain to gray matter and eight pertain to white matter.

**Table 4:** Various indicators of each model’s training set and test set.

model_name	Accuracy	AUC	95% CI	Sensitivity	Specificity	PPV	NPV	Precision	Recall	F1	Threshold	Task
LR	0.648	0.701	0.6412–0.7610	0.536	0.753	0.673	0.632	0.673	0.536	0.597	0.524	Train
LR	0.704	0.700	0.5756–0.8250	0.529	0.865	0.783	0.667	0.783	0.529	0.632	0.574	Test
SVM	0.782	0.861	0.8179–0.9045	0.761	0.801	0.784	0.780	0.784	0.761	0.772	0.490	Train
SVM	0.718	0.744	0.6247–0.8634	0.794	0.649	0.675	0.774	0.675	0.794	0.730	0.447	Test
KNN	0.634	0.785	0.7357–0.8345	0.304	0.945	0.840	0.590	0.840	0.304	0.447	0.600	Train
KNN	0.606	0.719	0.6014–0.8358	0.324	0.865	0.687	0.582	0.687	0.324	0.440	0.600	Test
RandomForest	0.757	0.828	0.7806–0.8745	0.746	0.767	0.752	0.762	0.752	0.746	0.749	0.466	Train
RandomForest	0.704	0.757	0.6455–0.8681	0.500	0.892	0.810	0.660	0.810	0.500	0.618	0.554	Test
ExtraTrees	0.715	0.768	0.7149–0.8220	0.674	0.753	0.721	0.710	0.721	0.674	0.697	0.482	Train
ExtraTrees	0.690	0.725	0.6064–0.8443	0.706	0.676	0.667	0.714	0.667	0.706	0.686	0.480	Test
XGBoost	0.842	0.919	0.8871–0.9509	0.841	0.842	0.835	0.848	0.835	0.841	0.838	0.489	Train
XGBoost	0.718	0.733	0.6134–0.8533	0.676	0.757	0.719	0.718	0.719	0.676	0.697	0.505	Test
LightGBM	0.785	0.863	0.8210–0.9047	0.775	0.795	0.781	0.789	0.781	0.775	0.778	0.481	Train
LightGBM	0.746	0.776	0.6636–0.8881	0.735	0.757	0.735	0.757	0.735	0.735	0.735	0.460	Test
MLP	0.704	0.772	0.7189–0.8257	0.804	0.610	0.661	0.767	0.661	0.804	0.725	0.442	Train
MLP	0.676	0.704	0.5810–0.8276	0.794	0.568	0.628	0.750	0.628	0.794	0.701	0.404	Test

LR, Logistic Regression; SVM, Support Vector Machines; KNN, K Nearest Neighbors; Extra Trees, Extremely Randomized Trees; XGBoost, eXtreme Gradient Boosting; LightGBM, Light Gradient Boosting Machine; MLP, Multilayer Perceptron; CI, confidence interval; PPV, Positive Predictive Value; NPV, Negative predictive value.

### Model efficiency comparison

In the test set, the LightGBM model achieved the highest AUC. The DeLong’s test revealed that although there is a difference between the AUC of the LightGBM model and other models, there is no statistically significant difference in performance (*p* > 0.05) compared to other models. In the training set, the AUC of the LightGBM model was ranked second, and the DeLong test showed that the performance of the LightGBM model was significantly higher than that of the other seven models (*p* < 0.001). Although the XGBoost model has the highest AUC in the training set, there is a significant difference compared to the AUC in the test set. Considering robustness, the LightGBM model is considered the most suitable model. For detailed information, please refer to Supplementary Table S2.

## Discussion

This study independently extracted imaging features from the gray and white matter of the cerebellum, creating a combined model of cerebellar gray and white matter imaging capable of objectively diagnosing MCI and HC. Among the models established by eight machine learning algorithms, the LightGBM prediction model emerged as the optimal choice for distinguishing between MCI and HC. The training set showed an AUC of 0.863 and an accuracy of 78.5%, while the validation set displayed an AUC of 0.776 and an accuracy of 74.6%. Notably, within the 16 imaging features of the LightGBM prediction model, the GM_wavelet_HLH_glrlm_RunVariance feature carried the highest weight.

The cerebellum, a brain structure primarily known for its role in motor functions, has recently gained attention for its involvement in cognitive functions (Piccinin et al., 2014; Weier et al., 2014). The cerebellar gray matter, located beneath the cerebellar cortex and consisting of neuronal cell bodies and dendrites, is an important component of the cerebellum. Studies using fMRI have confirmed that certain areas of the cerebellum are involved not only in motor control but also in cognitive and emotional functions (Sokolov et al., 2017; Guell et al., 2018). Beneath the gray matter is the white matter, which contains numerous nerve fiber bundles and several pairs of deep cerebellar nuclei responsible for transmitting information within the cerebellum and between the cerebellum and other brain regions (Prevosto et al., 2009). Neuroimaging studies have shown that the cerebellum forms anatomical connections with the prefrontal cortex, which are crucial for normal cognitive function (Gelinas et al., 2014; Buckner et al., 2011).

Research by Toniolo et al. found that patients in the MCI stage already exhibit atrophy of cerebellar gray matter, primarily in certain areas of the anterior and posterior lobes (Toniolo et al., 2018). Wang et al. discovered that first-order texture features from quantitative susceptibility mapping successfully distinguish AD and MCI from CN (Hwang et al., 2016). Radiomics is a method that extracts a large number of quantitative features from medical images to reveal underlying biological information and pathological changes. These features are particularly important in medical image analysis because it can quantitatively describe the characteristics of lesions, aiding in diagnosis, prognosis assessment, and evaluation of treatment response. These features have been proven to reflect the pathological changes in neurodegenerative diseases (Feng et al., 2018; Sun et al., 2018).

Pathological studies have confirmed Aβ amyloid deposition in the cerebellum of MCI patients (Mufson et al., 2011). Studies have shown that texture features of the hippocampus and corpus callosum can be used through machine learning to differentiate patients with MCI from healthy controls (Du et al., 2023; Shaji et al., 2022), based on high-throughput radiomic features numerically interpreting existing pathological changes. Therefore, the feasibility of this study’s model establishment is supported by both the pathological changes in the MCI cerebellum and the applicability of radiomics. When pathological changes occur in brain tissue, the texture of MR images may change accordingly (Sorensen et al., 2016; Chen et al., 2024). Based on this principle, we speculate that there may be certain imaging markers that can well reflect the pathological changes of MCI. This study combines radiomics with machine learning, using 3D T1WI images, focusing on the gray and white matter of the cerebellum, and establishes an efficient and objective differential model to distinguish MCI from NC.

The LightGBM algorithm is a tree-based gradient boosting algorithm known for its efficient reduction in memory consumption and computational complexity (Yanagawa et al., 2024). By utilizing a histogram-based decision tree algorithm, it significantly enhances training speed and efficiency. It’s most notable advantages include faster training speed, higher efficiency, and improved accuracy. In the LightGBM prediction model with 16 radiomic features, there are eight features related to gray matter and eight features related to white matter. The eight gray matter features include five GLRLM features, one GLCM feature, and two GLSZM features. Similarly, the eight white matter features consist of four first-order features, two GLSZM features, one shape feature, and one NGTDM feature. Among these, GM_HLH_glrlm_RunVariance is the most important feature. RunVariance refers to the variance of sequences where the same grayscale value appears consecutively in an image (Jin et al., 2023), reflecting the uniformity of image texture; a larger variance indicates a more uneven texture. In this study, when the Run Variance of the MCI group is smaller than that of the HC group, it may indicate that the texture within the cerebellar gray matter region of the MCI group is more uniform or has higher consistency, suggesting fewer local structural changes. The reduction in Run Variance may be associated with atrophy of the cerebellar gray matter (Toniolo et al., 2018; Kim et al., 2021). As patients with mild cognitive impairment may experience slight neuronal loss or reduced density of cerebellar gray matter, there would be fewer local structural changes, thus resulting in more uniform imaging texture features (Toniolo et al., 2018; Kim et al., 2021; Thomann et al., 2008). The decrease in Run Variance may also reflect a reduction in the microstructural complexity of the cerebellar gray matter. These identified abnormalities are closely associated with the underlying pathological changes in the cerebellum of MCI patients.

Other radiomics features also played a role in the established discriminant model, reflecting abnormal pathological changes in the cerebellar gray and white matter from different dimensions. It is noteworthy that we assumed the diagnostic efficacy of radiomics features extracted from the cerebellar gray and white matter for MCI to be the same. However, in the final model, the importance of gray matter seemed to be slightly higher. But this does not prove that the pathological changes in the cerebellar gray matter of MCI patients are more significant than those in the white matter. To address this question, it may be necessary to further expand the sample size and conduct multi-dimensional studies with more detailed disease progression classifications.

## Limitations

Our research has several limitations. Firstly, the data used in this study was obtained from the public database of ADNI and did not utilize internal data for further verification. Future studies should incorporate more centers to gather additional data for validation and optimization of the model. Secondly, this study solely employed imaging features to establish an independent imaging model, without further analyzing the relationship between these features and clinical scales or biological indicators. Thirdly, this study only conducted cross-sectional cohort comparison studies, without in-depth research on the role of these imaging features in the progression process. Fourth, we did not conduct a study on cerebellar involvement in patients with different subtypes of MCI. Lastly, current research has not utilized molecular biomarker data to further differentiate MCI caused by AD from MCI due to other etiologies, nor does it include longitudinal information on the conversion of MCI to dementia. This is worth exploring in future studies.

## Conclusion

To our knowledge, this is the first machine learning study to detect MCI based on cerebellar gray matter radiomics features. In this trial, a LightGBM machine learning model based on 16 radiomics features of cerebellar gray and white matter was able to accurately distinguish between MCI and HC based on conventional MRI images. Since this method is non-invasive, it has great potential for future clinical medical applications.

## Data Availability

The original contributions presented in the study are included in the article/Supplementary material, further inquiries can be directed to the corresponding author.

## Footnotes

^1^https://www.nitrc.org/projects/mricron/

^2^https://volbrain.upv.es/

^3^https://www.radiomics.io/pyradiomics.html
