# Supplementary material for: MRI radiomics combined with machine learning for diagnosing mild cognitive impairment: a focus on the cerebellar gray and white matter
Source: Front Aging Neurosci. 2024 Oct 4;16:1460293. doi: 10.3389/fnagi.2024.1460293 (PMC11489926; doi:10.3389/fnagi.2024.1460293)
Supplement: Supplementary file 1 [file Table_1.DOCX]

Supplementary Material

Figure


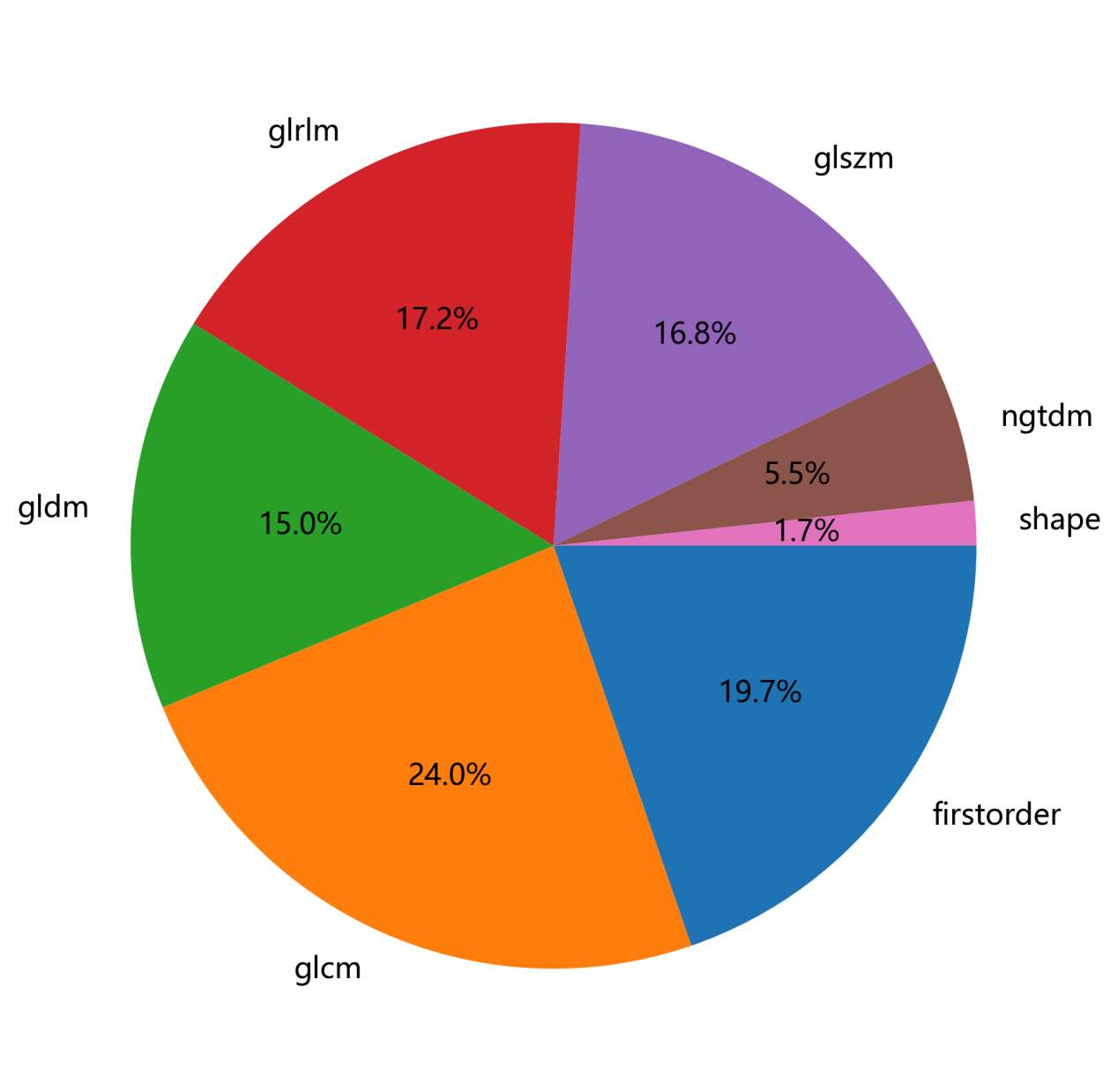


**Figure S1.** Proportional diagram of various radiomic features

**Materials and methods**

**Image Preprocessing and Radiomics Feature Extraction**

**Table S1. Detailed classification of radiomics features.**

| **Type** |  |  |  |  |  |
| --- | --- | --- | --- | --- | --- |
| First Order | Energy  Total Energy  Entropy  Minimum | 10th percentile  90th percentile  Maximum  Mean | Median  Interquartile Range  Range  Mean Absolute Deviation | Robust Mean Absolute Deviatio  Root Mean Squared  Standard Deviation | Skewness  Kurtosis  Variance  Uniformity |
| Shape | MeshSurface  PixelSurface  Perimeter  PerimeterSurfaceRatio  Sphericity  SphericalDisproportion | MaximumDiameter  MajorAxisLength  MinorAxisLength  Elongation  Compactness1  Compactness2 | MeshVolume  VoxelVolume  SurfaceArea  SurfaceVolumeRatio  Sphericity | SphericalDisproportion  Maximum3DDiameter  Maximum2DDiameterSlice  Maximum2DDiameterColumn  Maximum2DDiameterRow | MajorAxisLength  MinorAxisLength  LeastAxisLength  Elongation  Flatness |
| Grey Level Co-occurrence Matrix (GLCM) | Autocorrelation  ClusterProminence  ClusterShade  ClusterTendency  Contrast | Correlation  DifferenceAverage  DifferenceEntropy  DifferenceVariance  Id | Idm  Idmn  Idn  Imc1  Imc2 | InverseVariance  JointAverage  JointEnergy  JointEntropy  MaximumProbability | SumEntropy  SumSquares |
| Gray-level dependence matrix (GLDM) | DependenceEntropy  DependenceNonUniformity  DependenceNonUniformityNormalized  DependenceVariance  GrayLevelNonUniformity | GrayLevelVariance  HighGrayLevelEmphasis  LargeDependenceEmphasis  LargeDependenceHighGrayLevelEmphasis  LargeDependenceLowGrayLevelEmphasis | LowGrayLevelEmphasis  SmallDependenceEmphasis  SmallDependenceHighGrayLevelEmphasis  SmallDependenceLowGrayLevelEmphasis |  |  |
| Gray-level run length matrix (GLRLM) | GrayLevelNonUniformity  GrayLevelNonUniformityNormalized  GrayLevelVariance  HighGrayLevelRunEmphasis  LongRunEmphasis | LongRunHighGrayLevelEmphasis  LongRunLowGrayLevelEmphasis  LowGrayLevelRunEmphasis  RunEntropy  RunLengthNonUniformity | RunLengthNonUniformityNormalized  RunPercentage  RunVariance  ShortRunEmphasis  ShortRunHighGrayLevelEmphasis | ShortRunLowGrayLevelEmphasis |  |
| Gray-level size zone matrix (GLSZM) | GrayLevelNonUniformity  GrayLevelNonUniformityNormalized  GrayLevelVariance  HighGrayLevelZoneEmphasis  LargeAreaEmphasis | LargeAreaHighGrayLevelEmphasis  LargeAreaLowGrayLevelEmphasis  LowGrayLevelZoneEmphasis  SizeZoneNonUniformity  SizeZoneNonUniformityNormalized | SmallAreaEmphasis  SmallAreaHighGrayLevelEmphasis  SmallAreaLowGrayLevelEmphasis  ZoneEntropy  ZonePercentage | ZoneVariance |  |
| Neighboring gray tone difference matrix (NGTDM) | Busyness  Coarseness  Complexity  Contrast  Strength |  |  |  |  |
| Wavelet | HHH  HLH | HHL  HLL | LHH  LLH | LHL  LLL |  |

Note: Detailed meanings and formulas for each feature can be found at the URL (https://pyradiomics.readthedocs.io/en/latest/

Table S2. Comparison of Performance between LightGBM Model and Other Models

|  |  | LR | SVM | KNN | RandomForest | ExtraTrees | XGBoost | MLP |
| --- | --- | --- | --- | --- | --- | --- | --- | --- |
| LightGBM | Train | <0.001 | <0.001 | <0.001 | <0.001 | <0.001 | <0.001 | <0.001 |
|  | test | 0.075 | 0.407 | 0.290 | 0.634 | 0.271 | 0.227 | 0.072 |

**Results**

**Model Establishment**

After feature extraction is completed using Python code, we further use Python code to check for missing values. Through the inspection of the code, no missing values were found. This study extracts 833 radiomics features from each white and gray matter, including 14 shape features, 18 first-order features, 22 Gray-Level Co-occurrence Matrix (GLCM) features, 14 Gray-level dependence matrix (GLDM) features, 16 Gray level size zone matrix (GLSZM) features, 16 Gray level run-length matrix (GLRLM) features, 5 Neighboring gray tone difference matrix (NGTDM) features, and 728 wavelet features.

#code

import pandas as pd

# Load the CSV file into a DataFrame

file_path = 'your_file.csv' # Replace with the path to your CSV file

df = pd.read_csv(file_path)

# Check for missing values in each column

missing_values = df.isnull().sum()

# Print the results

print("Missing value check:")

for column, count in missing_values.items():

if count > 0:

print(f"{column} has {count} missing values")

else:

print(f"{column} has no missing values")

**Lasso model**

The specific parameters of the Lasso model can be referenced at https://scikit-learn.org/stable/modules/generated/sklearn.linear_model.Lasso.html?highlight=lasso#sklearn.linear_model.Lasso.

Rad_score = 0.4845-0.00001 × WM_original_glszm_GrayLevelNonUniformity

-0.0210 × WM_original_shape_LeastAxisLength

+0.0010 ×WM_wavelet_HLH_glszm_SmallAreaHighGrayLevelEmphasis

+0.0030 × WM_wavelet_HLL_firstorder_Maximum

+0.0087× WM_wavelet_HLL_firstorder_Skewness

-0.0021 × WM_wavelet_LLH_firstorder_RootMeanSquared

+0.0038 × WM_wavelet_LLL_firstorder_10Percentile

-0.0025 × WM_wavelet_LLL_ngtdm_Contrast

-0.0094 × GM_original_glrlm_RunEntropy

-0.0003 × GM_wavelet_HHH_glszm_SizeZoneNonUniformity

-0.0087 ×GM_wavelet_HLH_glrlm_RunEntropy

-0.0149 ×GM_wavelet_HLH_glrlm_RunVariance

-0.0141 × GM_wavelet_LHH_glrlm_RunEntropy

-0.0063 ×GM_wavelet_LHH_glszm_SizeZoneNonUniformity

-0.0004 ×GM_wavelet_LHL_glrlm_RunLengthNonUniformity

+0.0201 × GM_wavelet_LLL_glcm_ClusterShade
